# Advancements in Inlay Glenoid Components for Anatomic Total Shoulder Arthroplasty: A Review

**DOI:** 10.3390/jcm14165820

**Published:** 2025-08-18

**Authors:** Akshay R. Reddy, Keegan M. Hones, Taylor R. Rakauskas, Joseph J. King, Thomas W. Wright, Bradley S. Schoch, Kevin A. Hao

**Affiliations:** 1 College of Medicine, University of Florida, Gainesville, FL 32610, USA; 2 Department of Orthopaedics and Sports Medicine, University of Florida, Gainesville, FL 32607, USAhaoka@ortho.ufl.edu (K.A.H.); 3 College of Medicine, Florida Atlantic University, Boca Raton, FL 33431, USA; trakauskas2022@health.fau.edu; 4 Department of Orthopedic Surgery, Mayo Clinic, Jacksonville, FL 32224, USA

**Keywords:** inverted shoulder, total shoulder arthroplasty, inset glenoid, inlay glenoid

## Abstract

While anatomic total shoulder arthroplasty is a successful procedure that provides reliable pain relief and restoration of function in most patients, its success has been limited by glenoid component loosening. While series reporting the outcomes of inlay glenoid components have demonstrated excellent clinical outcomes with low rates of component loosening and need for revision, surgeons have been hesitant to adopt these implants due to concerns of inadequate pain relief secondary to the remaining glenoid rim contacting the humeral head implant. The inset glenoid component, a variant of the traditional inlay components, has gained interest because its design aims to achieve similar stability to traditional inlay components through implantation within strong subchondral bone, reduce the amount of glenoid vault removed compared to inlay components, and has a glenoid face designed to limit the rocking-horse phenomenon. In limited series, the inset glenoid component has demonstrated superior biomechanical and clinical performance compared to traditional onlay glenoid components. Although there have been minimal clinical studies investigating the inset glenoid in comparison to onlay and inlay components to date, a subset of case series with short-term follow-up have demonstrated favorable outcomes. The purpose of this article was to review the design rationale, biomechanical evidence, and clinical performance of the inset glenoid component.

## 1. Introduction

Anatomic total shoulder arthroplasty (aTSA) is a reliable treatment for various shoulder pathologies, including primary glenohumeral osteoarthritis (GHOA) and GHOA secondary to recurrent dislocation or osteonecrosis [1,2]. Whereas reverse total shoulder arthroplasty (rTSA) modifies shoulder biomechanics and shifts the reliance of motion and stability to the deltoid muscle, aTSA relies on the stabilizing force of the rotator cuff, similar to a native shoulder, and is thus not typically preferred in patients with rotator cuff pathology [3].

Since the first use of a glenoid prosthesis by Dr. Charles Neer decades ago, there have been several advancements in glenoid component design [4]. Yet, glenoid loosening remains a major limitation to aTSA survivorship, with the prevalence of radiographic aseptic loosening very high in series with long-term (15–20 year) follow-up [5]. In contrast, rTSA appears to achieve better time-zero glenoid fixation and lower rates of subsequent loosening, but often at the cost of poorer axial rotation and the risk of devastating complications such as fractures of the acromion and scapular spine [6,7]. While achieving a more anatomic reconstruction is attractive to many shoulder surgeons, future utilization of aTSA may remain somewhat restricted in certain populations until glenoid component loosening and associated rotator cuff failure are reduced. Theoretically, the proposed benefit of the inset glenoid is that it would require less lateralization, thus easier repair of the subscapularis, and reduced risk of edge loading and premature loosening [8,9].

**Figure 1:**
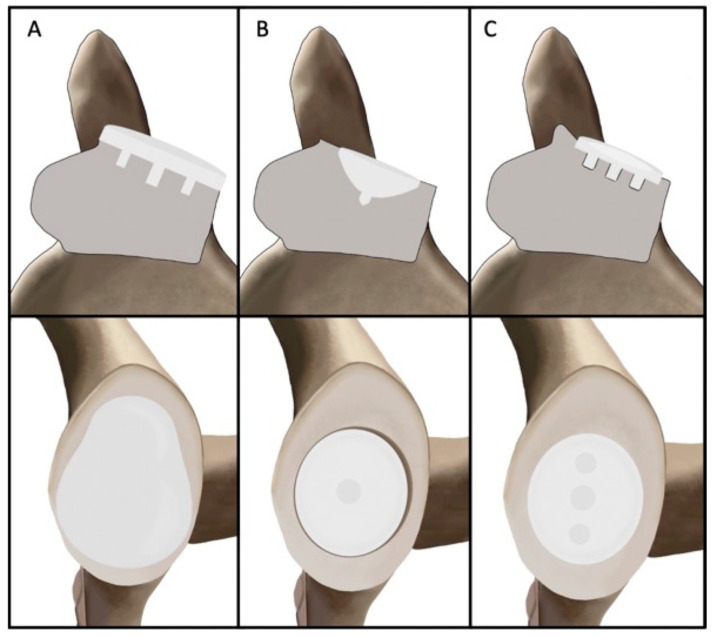
Glenoid designs in anatomic total shoulder arthroplasty can be classified according to their interface with glenoid bone as onlay (**A**) or inlay (**B**). The new Inset glenoid includes characteristics of both onlay and inlay glenoid components with one central peg and two peripheral pegs (**C**).

Contemporary aTSA glenoid components can be classified according to their interface with glenoid bone as onlay or inlay (Figure 1). Traditional onlay glenoids cover the entire glenoid articular surface and require minimal reaming of native bone. However, studies have demonstrated a “rocking horse phenomenon” which occurs secondary to edge loading of the onlay component, which has led to the development of inlay glenoid components, which are completely surrounded by bone and have greater peripheral support and load-sharing [9,10]. Although inlay glenoid components have shown lower rates of glenoid loosening and a favorable biomechanical profile compared to onlay glenoids, inlay glenoids may require removal of more glenoid subchondral bone and glenoid vault [8,11]. This limitation has led to the development of the inset glenoid, which was designed to minimize edge loading by seating the component partly within a reamed recess surrounded by native bone while relatively preserving the glenoid vault [12]. The purpose of this article was to review the design rationale, biomechanical evidence, and clinical performance of the inset glenoid component and highlight potential comparisons to traditional inlay and onlay designs.

## 2. Design Philosophy

Dr. Neer’s original TSA design included a humeral stem and a cemented, all-polyethylene (PE) keeled glenoid component. From Neer’s initial results, subsequent generations of all-PE prostheses arose, including both conforming designs, which include an artificial labrum, and non-conforming designs, both aimed at improving glenohumeral stability. However, with no radial mismatch, studies on these constrained components showed increased backside radiolucency and contact stress [13]. Replication of the native glenoid’s shape and concavity has been seen to be inferior in some aspects to elliptical designs in aTSA, potentially due to the arthritic glenoid’s non-anatomic shape and the better fit of properly sized elliptical implants into the glenoid after reaming [14,15,16]. However, while elliptical designs may lower peak contact pressure (PCP) on the humeral side [17], it has been shown that they may lead to more glenohumeral translation on the glenoid side and micro-motion of the glenoid component [18].

In addition to the glenoid face concavity, the ideal backside geometry of the glenoid component has also been considered; studies have demonstrated that curved designs resist translations and have better radiolucency scores compared to flat designs [19]. The traditional keeled design introduced by Neer resembles a “shark fin” and incorporates a rectangular-like base [20]. Subsequently, a pegged design was introduced that included several pegs with the purpose of utilizing stronger peripheral bone for fixation, and has demonstrated radiographic superiority to its keeled counterpart in some studies [21,22,23,24]. Recent studies have shown improved rates of revision in crosslinked polyethylene (XLPE) and modern fixation methods of glenoid components [25,26]. Page et al. demonstrated that crosslinked polyethylene (XLPE) should be preferred over non-XLPE as well [26]. In addition, Dillon et al. demonstrated that central-pegged ingrowth designs have a significantly lower rate of revision in comparison to cemented pegged and keeled glenoid [25].

In addition to all-PE glenoid components, metal-backed and hybrid components have also been used. However, metal-backed glenoids have fallen out of favor due to high rates of failure, with a rate of revision over three times greater compared to all-PE designs [27]. In light of the failure of metal-backed glenoids, hybrid glenoids were designed with the aim of achieving both the time-zero fixation provided by cementing all-PE components with the long-term advantage of biologic in-growth of metal components [28,29,30,31,32]. While low rates of complications and revision surgery have been reported for hybrid components at early follow-up, their long-term performance remains to be determined.

In addition to evaluating different geometric and component material properties, the placement of the backside of the glenoid component with respect to the glenoid surface has also been evaluated. Traditional onlay glenoid designs sit on top of the glenoid face and require minimal subchondral reaming of native bone to create a curved surface to mate with the backside of the implant. The design rationale of the onlay glenoid was to replicate the sagittal plane geometry of the anatomic glenoid concavity. Although onlay glenoids are very commonly used, they are prone to loosening due to edge loading during humeral head translation on the glenoid, coined the “rocking horse” phenomenon [8,13]. The phenomenon has been observed in both keeled and pegged components. Inlay glenoid components were developed in response to failures seen with onlay components. Inlay components are placed completely flush with the glenoid face, which has been shown to decrease the rocking horse phenomenon during shoulder motion [8,11]. The backside of the inlay design most often utilizes a central peg; however, other inlay designs sometimes include more pegs, such as in the FX Shoulder USA varieties (FX Shoulder USA Inc., Dallas, TX, USA). Although inlay glenoids have demonstrated durable outcomes in challenging glenoid bone loss patients with low rates of loosening [10,33], their use has been limited to patients with severe glenoid retroversion due to many surgeons’ hesitancy with removing substantial portions of the glenoid vault.

**Figure 2:**
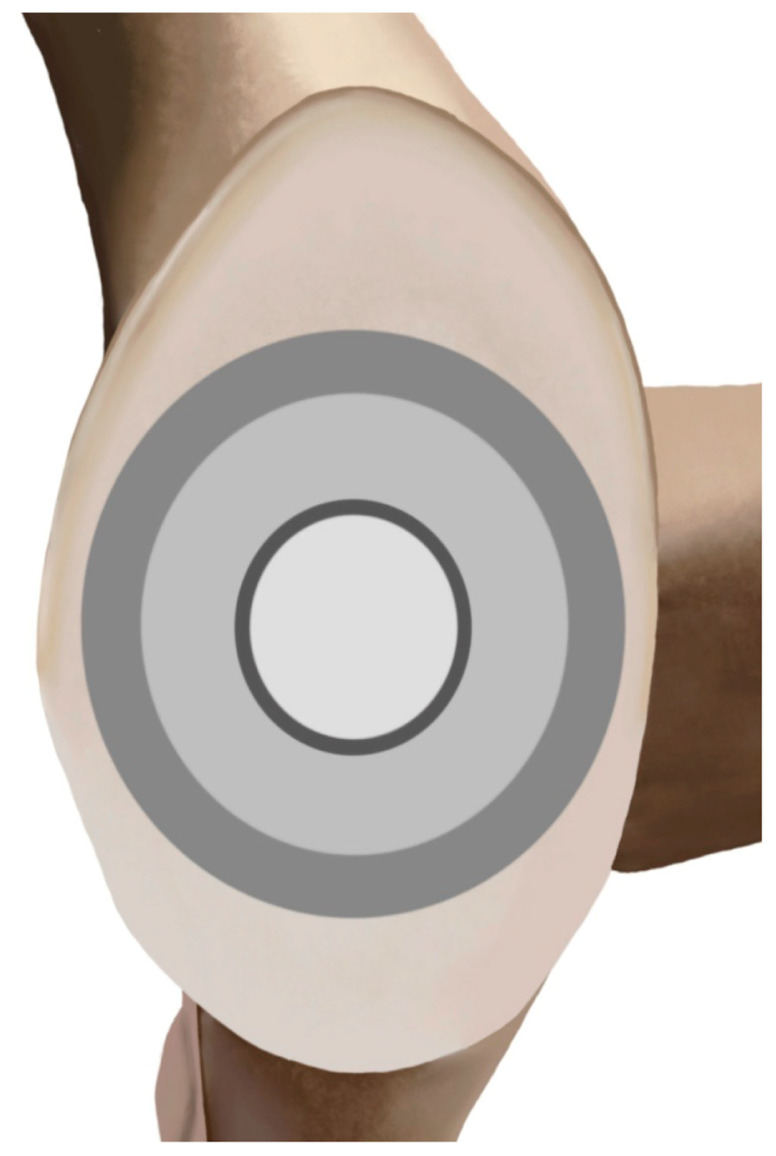
The inset glenoid is comprised of three different zones [40]. The central zone provides contact with the polyethylene at a location where the humeral head is typically placed at rest. The second zone is designed to conform during abduction. The outer zone provides a larger radial mismatch for when the humeral head translates without catching the ends of the implant, a typical issue in onlay glenoids.

The inset glenoid was designed as an alternative to onlay and inlay glenoid components for patients with severe GHOA and glenoid bone loss. The design includes the incorporation of three different zones on the backside of the component, designed to minimize the “rocking horse” phenomenon (Figure 2) [12]. The designers of the inset glenoid theorize that the central zone provides contact where the humeral head component typically lies, the second zone provides conformation to the humeral component during abduction, and the outer zone provides a larger radial mismatch for when the humeral head translates without catching the ends of the implant, a common issue seen with onlay glenoids. Importantly, the inset glenoid component, which can be classified as an inlay design, differs from “inset” implantation of an onlay glenoid component. Inset by technique is the implantation of a traditional onlay component within a partially-reamed concavity [34]. However, given that inset designs are smaller than a native glenoid, it is a concern that inset implants may not be practical in larger patients. The downside of incomplete peripheral coverage may supersede the inset’s proposed benefit of reducing edge loading, a key component of the inset glenoid’s design [35,36,37]. Further, the inset design includes cementing of the posterior peg and of multiple small drilled holes in the subchondral bone to supplement cement fixation. In addition, cement was applied and pressed to the back of the implant to fill the cement channels, with excess cement being subsequently removed [38,39].

Unlike traditional inlay glenoid components, whose implantation requires reaming through subchondral bone, the aim of the inset component is to minimize the need for reaming and instead utilize strong subchondral bone for fixation, which has been shown to have greater thickness in arthritic patients compared to subchondral bone in healthy patients [41,42]. The design of the inset glenoid theoretically takes advantage of this unique feature of surface bone, as it is essentially a disc inset into the strong surface, analogous to a manhole on the road [12]. Second, the circular glenoid component is implanted partly within a reamed recess and surrounded by native bone. This feature may decrease the amount of bone removal and minimize edge loading [38]. Lastly, the inset component does not have several long pegs, as the support from the native bone at the rim is thought to reduce the need for backside fixation deep within the glenoid vault [12]. Rather, the backside of these implants consists of a single central peg and two cement channels to resist the breaking of the cement mantle.

## 3. Biomechanics Evidence

Biomechanical studies have demonstrated reduced edge loading and loosening with the use of inlay compared to onlay components. In 2017, Gagliano et al. compared the fixation strength and contact forces between an inlay glenoid (Ovo system; Arthrosurface, Franklin, MA, USA) and an onlay glenoid component (DonJoy; Turon system, DJO Global/Enovis, Wilmington, DE, USA) [8]. Measurements were made using the TekScan (Tekscan, Norwood, MA, USA) pressure sensor before and after implantation and at the anterior and posterior edges of the implants, with each model tested over 4000 cycles. The mean surface contact forces for the onlay glenoid edge were seen to be greater than the inlay implants. Further, fatigue testing revealed significantly decreased loosening of the inlay glenoid [23].

The first biomechanical evaluation of the inset glenoid component was performed by Gunther et al. in 2012 [9]. The component was a circular glenoid implant with a 35 mm diameter and one 8 mm central peg. Humeral head diameters included 38 mm or 56 mm. In the first part of the study, these configurations were compared to standard 40 mm keeled and pegged onlay glenoid components (DePuy, Warsaw, IN, USA) under testing conditions of 100,000 cycles at 750 Newtons of force applied to the glenoid according to the Anglin et al. and ASTM protocol [43]. The authors found no glenoid implants to display signs of loosening [9]. However, a significant reduction in post-test distraction, the displacement of the glenoid component away from the testing surface, was seen with the inset glenoid in comparison to both the pegged and keeled onlay glenoids. The second portion of the study was the finite element analysis, which characterized polymer displacement along the articular surface and backside of the implant under rim loading conditions. The inset glenoid was found to experience less stress in the central region of the implant in comparison to the pegged onlay glenoid and less stress in the peripheral zones compared to both pegged and keeled onlay glenoids. An 87% and 73% reduction in displacement was seen with the inset glenoid compared to the onlay pegged and keeled designs, respectively. Recently, Khan et al. demonstrated that circular glenoids with peripheral ring fixation display increased bone preservation and reduced “rocking horse phenomenon” in comparison to standard pegged designs as well [44]. The periphery of native bone is believed to increase the strength of fixation and limit the need for extensive pegged or keeled implantation into the glenoid [12]. Although these favorable biomechanical results of the inset glenoid are promising, future biomechanical studies comparing the inset glenoid to traditional inlay glenoids are needed, along with a greater variety of humeral head diameters under similar testing conditions. Further, it is unclear how utilizing an inset technique with an onlay glenoid compares to the inset glenoid component.

## 4. Clinical Evidence

Studies have demonstrated the success of inlay glenoids in treating various pathologies such as GHOA, severe glenoid dysplasia, and glenoids with severe bone loss (Table 1) [10]. Berk et al. conducted a systematic review of five studies [10,45,46,47,48], totaling 148 shoulders with GHOA, treated with primary TSA with an inlay glenoid component [33]. The mean follow-up was 47.1 months (range, 41–81 months) across all studies. Mean visual analog scale (VAS) pain scores significantly decreased from 6.9 ± 2.4 to 1.6 ± 2.3 points, Penn shoulder score (PSS) increased from 27.6 ± 11.3 to 53.4 ± 6.1, the American Shoulder and Elbow Surgeon (ASES) score increased from 34.1 ± 19.4 to 80.6 ± 19.2, Single Assessment Numeric Evaluation (SANE) scores increased from 25.9 ± 22.2 to 80.7 ± 22.2, forward elevation (FE) increased from 110° to 156°, and ER improved from 22° to 51°. Complications included one (0.7%) postoperative hematoma [46], one (0.7%) pulmonary embolism [46], and one (0.7%) case of postoperative arthrofibrosis [48]. One patient (0.7%) had an intraoperative glenoid rim fracture, leading to advanced radiolucency [48]. Two revisions (1.4%) to rTSA were reported at 1.7 and 2.2 years postoperatively [10]. These results demonstrate that use of an inlay glenoid component is associated with improvements in postoperative pain, function, and satisfaction while minimizing rates of glenoid component loosening and the need for revision surgery [27,49,50]. Additionally, the inlay glenoid has been shown to perform well in younger, active patients with a high rate of return to activity [46]. However, limitations to inlay glenoid use include the limited long-term evidence regarding survivorship and a technically difficult implantation, which can result in peripheral rim fracture [33]. Further, the inlay glenoid may demonstrate more restricted sizing, as many studies have reported not using the implant in patients with greater deformity or dysplasia [8,46].

In 2012, Gunther and Lynch reported the short-term outcomes of seven patients with glenoid bone loss (neutral glenoid vault depth <15 mm) secondary to primary GHOA who underwent aTSA using the inset glenoid [38]. Walch classifications of included shoulders were two A2, two B2, and three C glenoids. At an average of 4.3 years post-implantation, significant improvements in the ASES score (25.6 ± 12.1 to 94.1 ± 5.2, *p* < 0.02), VAS score (6.9 to 0.1, (*p* < 0.02), FE (increase of 33°), ER (increase of 34°), and IR (increase by six spinal levels) were reported (all *p* < 0.02). Radiographic assessment showed all the glenoid components were at “low risk” for glenoid loosening, characterized by a classification system used in prior onlay studies with points assigned based on the thickness of any lucent lines present [51,52]. As glenoid version and depth were measured preoperatively, a custom guide was utilized to correct the version and tilt of the glenoid. For severely deformed glenoids, partial correction of retroversion was accepted. The glenoid surface was reamed 2–3 mm, and the implant surface edge was left 2 mm above the sclerotic bone, lateralizing the joint in most cases or maintaining the joint line if the version was corrected. There were no perioperative complications or postoperative subluxation in any patient. Three patients showed no evidence of lucency. One patient had a 0.25 mm line in zone 2. Two patients had glenoid component lucent lines less than 1 mm in zones 2 and 4. One patient had a glenoid component lucent line less than 1 mm in zone 2.

In 2016, Davis et al. conducted the second short-term retrospective study evaluating outcomes of the inset glenoid in patients with severe glenoid dysplasia and/or bone loss [39]. Nine shoulders were included in the study with a minimum two-year follow-up. Glenoid deformities in the study included four Walch type A2, two Walch type C, and three unable to be classified. For all patients, the humeral head was resected using the patient’s normal retroversion; however, patients with more severe retroversion did not undergo posterior capsule or posterior glenohumeral ligament release. In severe cases (>45 degrees) of retroversion, the Arthrosurface component was used as the glenoid was able to be prepared at a 30-degree angle. Primary arthroplasty was performed on 6/9 shoulders, and revision arthroplasty was performed on 3/9 shoulders. At an average of 2.8 years post-implantation, significant improvements in SANE score (32% to 89%, *p* < 0.001), VAS score (8 to 1, *p* < 0.001), FE (112° to 160°, *p* < 0.001), and ER (28° to 42°, *p* < 0.001) were reported [39]. No clinical glenoid loosening was seen at a mean 2.8-year follow-up, as measured using CT tomography using the technique described by Freidman et al. [39,53]. No intraoperative complications or reoperations were reported.

In 2019, Gunther and Tran reported their long-term outcomes in 24 patients with severe GHOA treated with the inset glenoid [54]. Severe glenoid bone deficiency was characterized similarly to Gunther’s initial study, with a glenoid vault depth <15 mm [38,54]. The same techniques for correcting retroversion as in Gunther’s 2012 study were utilized. Similarly, a custom guide and reamer were used to create an inset pocket 2–3 mm into the bone rather than leaving the entire surface [38]. At an average of 8.7 years post-implantation, significant improvements in ASES score (23.0 ± 12.6 to 95.0 ± 3.9, *p* < 0.001), VAS score (7.7 ± 1.6 to 0.1 ± 0.3, *p* < 0.001), FE (95° to 131°, *p* < 0.001), ER (18° to 49°, *p* < 0.001), and IR (five spinal levels, *p* < 0.001) were reported [54]. Further, there was no observed glenoid loosening, evaluated using Gunther et al.’s classification system for inset glenoids described in 2012 [38]. This system divided the inset implant into five zones, with points added by the thickness of any lucent lines, with scores from 0 to 3 assigned for ≤1 mm, 1–1.5 mm, and >1.5 mm, respectively. Low risk for loosening was defined as 0–5 points, and high risk was 11–15 points or any implant tilt or subsidence. Six of the 21 patients had radiolucent lines at final follow-up with a maximum radiographic score of five, which is defined as “low risk” for implant loosening. There were also no surgical complications or revision surgeries performed [54].

The most recent clinical study investigating the inset glenoid component was published by Johnston et al. in 2023 [50]. Seventy-five shoulders were included in the final analysis at a mean follow-up of 2.4 years. Twenty-one of the glenoids were classified as Walch A1, 10 classified as Walch A2, 13 classified as Walch B1, 22 classified as Walch B2, six classified as Walch B3, and three were classified as Walch D. For all glenoids, a single pocket was reamed 2–3 mm to create a surrounding rim of cortical bone, and peripheral holes were subsequently drilled. At an average of 2.4 years post-implantation, significant improvements in ASES score (44 ± 18 to 87 ± 15, *p* < 0.001), VAS score (5.1 ± 2.7 to 0.9 ± 1.6, *p* < 0.001), SANE score (40 ± 25 to 91 ± 13, *p* < 0.001), FE (115° to 150°, *p* < 0.001), ER (30° to 53°, *p* < 0.001), and IR of L5 or higher (27/75 to 54/75, *p* < 0.001) were reported. Low rates of central peg lucency (5.3%) and glenoid loosening (1.3%) were reported. There was one case of glenoid loosening, characterized as tilting of the implant within the glenoid vault, reported for a patient with a Walch B3 glenoid. Additionally, there were four (5.3%) postoperative complications and three (4%) reoperations due to either inadequate subscapularis healing or acute rotator cuff tear. There was no revision performed for implant-related complications.

**Table 1:** Summary of clinical series in the literature reporting the outcomes of ATSA using the Inset glenoid component.

Study	Study Design	Implant	N (Shoulders)	Follow-Up (Mean, Minimum, Range)	Pre- to Postoperative Improvement in Outcomes (Mean ± SD Unless Otherwise Specified)	Complications
Gunther et al. (2012) [38]	Retro	Custom implants manufactured by Biomet, Inc. (Warsaw, IN, USA)	7	52 months, 36 months, 44–60 months	ASES score: 25.6 ± 12.1 to 94.1 ± 5.2 (*p* < 0.02). VAS score: 6.9 to 0.1 (*p* < 0.02). ROM: Increase in FE by 33 degrees, ER of 34 degrees, and an IR of 6 spinal levels (all *p* < 0.02).	One patient had a 0.25 mm line in zone 2, but no glenoid component lucent lines. Two patients had glenoid component lucent lines less than 1 mm in zones 2 and 4. One patient had a glenoid component lucent line less than 1 mm in zone 2. No perioperative complications or postoperative subluxation.
Davis et al. (2016) [39]	Retro	Ascension Orthopaedics [Austin, TX, USA] Shoulder Innovations [Ada, MI, USA] Arthrosurface [Franklin, MA, USA])	9	34 months, 36 months, 25–43	VAS score: 8 to 1 (*p* < 0.001). SANE score: 32% to 89% (*p* < 0.001). ROM: FE: 112° to 160° (*p* < 0.001). ER: 28° to 42° (*p* < 0.001).	One patient without follow-up at 24 months had a mild axillary nerve palsy. No intraoperative complications or reoperations were reported.
Gunther et al. (2019) [54]	Retro	Custom implants manufactured by Biomet, Inc. (Warsaw, IN, USA)	24	104 months, 72 months, 76–142 months	ASES score: 23 ± 12.6 to 95 ± 3.9 (*p* < 0.001). VAS score: 7.7 ± 1.6 to 0.1 ± 0.3 (*p* < 0.001). ROM: FE: 95° to 131° (*p* < 0.001). ER: 18° to 49° (*p* < 0.001). IR by five spinal levels (*p* < 0.001).	6/21 patients had radiolucent lines at final follow-up with a maximum radiographic score of 5. No surgical complications or reoperations.
Johnston et al. (2023) [50]	Retro	InSet glenoid implant (Shoulder Innovations, Grand Rapids, MI, USA)	75	28.7 months, 24 months, 24–42 months	ASES score: 44 ± 18 to 87 ± 15 (*p* < 0.001). VAS score: 5.1 ± 2.7 to 0.9 ± 1.6 (*p* < 0.001). SANE score: 40 ± 25 to 91 ± 13 (*p* < 0.001). ROM: FF: 114.5° to 150.1° (*p* < 0.001). ER: 29.8° to 53° (*p* < 0.001). IR, L5 or higher: 27/75 to 54/75 (*p* < 0.001).	One case of glenoid loosening (type B3 glenoid). Four postoperative complications. Three reoperations. No intraoperative complications.

SD, standard deviation; Retro, retrospective; ASES, American Shoulder and Elbow Surgeons; VAS, visual analog scale; ROM, range of motion; FE, forward elevation; ER, external rotation; IR, internal rotation; SANE, Single Assessment Numeric Evaluation.

## 5. Special Considerations

Although whether the inset glenoid can be safely used in a wide array of patients indicated for aTSA requires further clinical investigation, there may be a subset of patients that may particularly benefit from the inset design over a traditional inlay or onlay component. The inset glenoid may be promising for patients with small glenoid vaults that cannot safely be treated with onlay components because they are likely to perforate, compromising time-zero stability. Given the potential reduction in reaming needed for the inset glenoid compared to traditional inlay components, it may also be uniquely advantageous for young patients with significant glenoid deformity and pain that desire excellent ROM, which an rTSA may not reliably provide. In the given clinical studies presented (Table 1), the inset glenoid was implanted in patients with a perpendicular glenoid vault depth under 15 mm, indicating use in patients in which standard (onlay and inlay) components were not indicated. In addition, inset components may offer the benefit of simpler fixation and less bone removal in comparison to their inlay counterpart, as there is no need for extended pegs, which may prove beneficial [12]. However, more limited sizing options may potentially limit use in certain populations [40].

However, more studies are needed before widespread adoption of the inset in patients with GHOA and sufficient vault volume, as well as patients with other diagnoses such as atraumatic avascular necrosis. Further, other options such as keeled implants have been mentioned, though their long-term survival at 10 years has been shown to be limited, trimming the peripheral pegs of an onlay glenoid for small glenoids, or even the ream and run technique could be considered by surgeons [55].

## 6. Conclusions

Inlay glenoid components have gained interest in the field of shoulder arthroplasty and may present an opportunity for surgeons to achieve a more anatomic reconstruction with reduced risk of glenoid component loosening. The inset glenoid component reduces the removal of native glenoid bone stock and utilizes the stronger subchondral bone, which contrasts with the flush fit of traditional inlay glenoid components that require substantial removal of glenoid vault. Biomechanical studies of the inset glenoid have demonstrated substantially reduced edge loading and “rocking horse” phenomenon compared to onlay components. A subset of case series with short-term follow-up has demonstrated significant improvement in pain scores, ROM, and has shown limited to no glenoid loosening or need for reoperation. However, future studies comparing inset glenoids directly to inlay glenoids are warranted to better understand the outcomes of these new components.
